# What if Horses Were Humans? Comparing Rein Tension and Bit Pressures to Human Pressure Pain Thresholds

**DOI:** 10.3390/ani15202989

**Published:** 2025-10-15

**Authors:** Frauke Musial, Thomas Weiss

**Affiliations:** 1 National Research Center in Complementary and Alternative Medicine (NAFKAM), Department of Community Medicine, Faculty of Health Sciences, UiT The Arctic University of Norway, 9037 Tromsø, Norway; 2 Department of Clinical Psychology, Institute of Psychology, Friedrich Schiller University Jena, 07743 Jena, Germany

**Keywords:** horse, human, pressure pain, equestrian sports, bit pressure, rein tension, pressure pain detection thresholds, stimulus response function, animal welfare

## Abstract

**Simple Summary:**

Potential bit pressure and rein tension-induced mouth pain in horses has become a significant welfare concern, sparking debates within both the equestrian community and the general public. Evidence suggests that bits can cause pain-related behaviors in horses and even lesions within the oral cavity. While the psychophysiology and psychophysics of pressure-induced pain are well understood in humans, conducting similar studies in animals presents challenges. However, there is a broad consensus that the physiology of pain processing is similar across mammals. Therefore, it is reasonable to assume that pain perception in humans and horses is principally comparable. In this context, we compare established human pressure pain detection thresholds (PPDTs) for the face, hand, and foot with bit pressures and rein tensions reported in the scientific literature. This comparison reveals that only the lowest reported pressures would likely be pain-free for humans. By contrast, average to strong or maximum pressures would induce severe pain sensations if applied to the face, hands, or feet of humans. In conclusion, bit pressures as reported in the scientific literature would likely induce strong pain sensations in humans, raising significant concerns about horse welfare.

**Abstract:**

Bit pressure and rein tension-induced mouth pain in horses have recently become a significant welfare concern, fueling debates within the equestrian community and beyond. Evidence indicates that bits can cause pain-related behaviors and even oral lesions. Although studying pressure-induced pain in animals is challenging, the similarities in the physiology of pain processing (nociception) across mammals suggest that it is reasonable to assume that pain perception in humans and horses is principally comparable. Therefore, we compared human pressure pain detection thresholds (PPDTs) to reported rein pressures in equestrian sports as reported in the scientific literature. Reported rein tensions (kPa) range from a minimum of 91.2–107.87 to a maximum of 1314.09–4285.51, while human PPDTs (in kPa) are 232.4 for the face, 445.3 for the hand, and 535.5 for the foot. These comparisons reveal that only the lowest reported bit pressures would be pain-free for humans. Average to maximum pressures would cause strong to severe pain sensations in humans. Furthermore, data on pressure pain-induced stimulus response functions suggest that bit pressures commonly encountered in equestrian sports could cause lesions in humans, making them unacceptable in human experimentation. In conclusion, bit pressures as reported in the scientific literature would cause significant pain if applied to humans, raising welfare concerns for horses.

## 1. Introduction

Bit pressure and rein tension-related mouth pain and injuries in horses have become a growing concern in equitation science over the past decade, with heightened attention in recent years. The issue recently reached a critical point when several researchers penned an open letter of concern to the Fédération Equestre Internationale (FEI). In a video presentation to the FEI on the 9th of April 2025, Wilkins et al. [1] summarize and explain the scientific evidence that bits work because they inflict considerable pain and why horses are still capable of performing even under these conditions. This letter, combined with other reports of abuse and threats to animal welfare in equestrian sports, has sparked a public and, at times, emotionally charged debate about the social license of equestrian sports [2].

While there is substantial evidence that bits can cause lesions in a horse’s mouth (e.g., Refs. [3,4,5,6,7,8,9]) and the welfare implications of these findings are extensively discussed elsewhere, it remains unclear whether these findings represent a systemic issue inherent to the use of bits in equestrian sports or whether they are specifically linked to a practice of applying extreme rein tension to a bit in the horse’s mouth. Nevertheless, the existing evidence of bit-induced lesions is compelling and raises significant welfare concerns (for an overview, see Mellor [10].

To further complicate the situation, horses lack a specific vocalization to express pain and, as prey animals, tend to mask their discomfort to the greatest extent possible. Torcivia and McDonnell [11] found that hospitalized horses with pain decreased their discomfort behavior during caretaker visits. This creates further welfare challenges, as identifying pain in horses is significantly more difficult compared to other companion animals, such as dogs.

Regarding the use of bits to control horses but also with respect to the associated welfare concerns, it is crucial to determine whether it is principally possible to use a bit without causing pain. To explore this question, it would be necessary to establish the pressure pain detection threshold (the lowest intensity at which a pressure stimulus is perceived as painful) on a horse’s tongue and other oral tissues. However, conducting such elaborate psychophysical experiments is not feasible in non-human animals.

Nonetheless, horses are mammals, and mammals share many fundamental physiological functions. There is broad consensus that the physiology of pain processing, nociception, is also similar across mammals [12,13,14,15]. However, the phenomenon of “pain” is more complex and is often accompanied by intense emotions. The International Association for the Study of Pain (IASP) defines pain as “an unpleasant sensory or emotional experience that is associated with or resembles actual or potential tissue damage” Raja et al. [16].

Regarding similarities in nociception, the pain detection threshold is generally expected to remain relatively consistent across the mammalian realm, although variations may occur. In contrast, pain tolerance may be more closely tied to a species’ lifestyle, habitat, and behavioral necessities, and may therefore be more variable between species. Given the similarities in nociception among mammals [12,13,14,15], we believe it is reasonable to assume that pain detection thresholds in humans and horses are fundamentally comparable. Moreover, a recent study conducted a “Comparative Neuro-Histological Assessment of Gluteal Skin Thickness and Cutaneous Nociceptor Distribution in Horses and Humans” [17]. This study clearly demonstrated that the superficial pain-sensitive epidermal layer of horse skin is as richly innervated as and of equivalent thickness to the human skin. This finding confirms that humans and horses possess equivalent basic anatomical structures for detecting cutaneous pain and further supports the assumption of a fundamentally similar pain processing mechanism between humans and horses. However, it is important to consider that the experience of pain and its regulation may differ between humans and horses, particularly due to the horse’s nature as a flight animal.

Thus, the aim of this commentary is to relate the existing knowledge about the psychophysiology/psychophysics of human pressure pain to rein tensions and bit pressures occurring in equestrian sports as reported in the scientific literature. Our approach seeks to interpret existing knowledge within the context of the phylogeny of nociception and pain processing in mammals, focusing on two species—the horse and the human—that share a common history within the framework of domestication.

## 2. Pressure Pain Detection Threshold

Pressure pain detection thresholds (PPDTs) in humans are well-established, particularly through a measurement system called “quantitative sensory testing” (QST), which is a widely used and highly reliable methodology in human pain research [18,19,20,21,22]. Furthermore, the standard measurement points for QST typically include the foot, hand, and face. Additionally, normative data for the human back are available [22]; however, these data are not considered highly relevant in this context. Nonetheless, they may become pertinent when discussing pressure pain caused by ill-fitting saddles or the impact of heavy riders (in comparison to the horse).

Although there is currently no normative QST data available for pressure-induced pain in the human tongue or other oral cavity tissues, the QST normative data for the human face remain highly relevant for understanding the potential pain caused by bit pressure in horses. PPDTs for facial areas can serve as a conservative estimate of pain perception in the tongue or other oral cavity tissues. Nonetheless, it is likely that pain detection thresholds are considerably lower for the tongue or the oral cavity as a whole [23].

Mellor’s [10] thorough review offers a comprehensive summary and classification of rein tensions discussed in the literature [10] (p. 7). His considerations are based on the assumption that bridles are typically adjusted to position the bit in contact with a mostly toothless area of the gums on either side of the mandible, located between the incisors and premolars in what is known as the “interdental space”, an area densely populated with nociceptors ([23,24,25,26,27], as referenced in Mellor [10], p. 7).

To illustrate the forces associated with rein tension acting on the bit in a horse’s mouth, Mellor recalculates the forces reported in Newtons (N) into mass per unit area, using an estimated bit-gum contact area. The recalculated data are then converted and presented as kg/cm^2^, a format that facilitates comparison with human pressure pain thresholds, which are typically expressed in kilopascals (kPa)—another unit of mass per unit area. In his 2020 review, Mellor [10] presents three summarized categories of bit-related pressures (also referred to as rein tension) based on ranges derived from the scientific literature: overall mean, maximum values, and minimum values. Notably, the minimum values are based on data from a single study conducted by Heleski et al. [28]. We will use the values provided by Mellor [10] (p. 7) in this context (see Table 1).

Average human PPDTs were calculated for three different locations—face, hand, and foot—using the normative data provided by Magerl et al. [20]. Magerl et al. [20] reported values separately for five different age groups (20–30, 30–40, 40–50, 50–60, >60) as well as by gender. For the purposes of this publication, the mean value for each location (face, hand, and foot) was calculated across all age groups and genders. The original data, presented in kilopascals (kPa), are provided here in both kilopascals (kPa) and kilograms per square centimeter (kg/cm^2^). Given the research objective—to investigate human PPDTs in the context of potential bit-induced mouth pain in horses—the facial PPDT was identified as the most relevant measure. The summarized normative human PPT data [20] are presented in Table 2. As anticipated, the PPDT for the face—a bony structure with densely innervated skin—is significantly lower compared to the PPDTs for the hand or foot.

**Figure 1:**
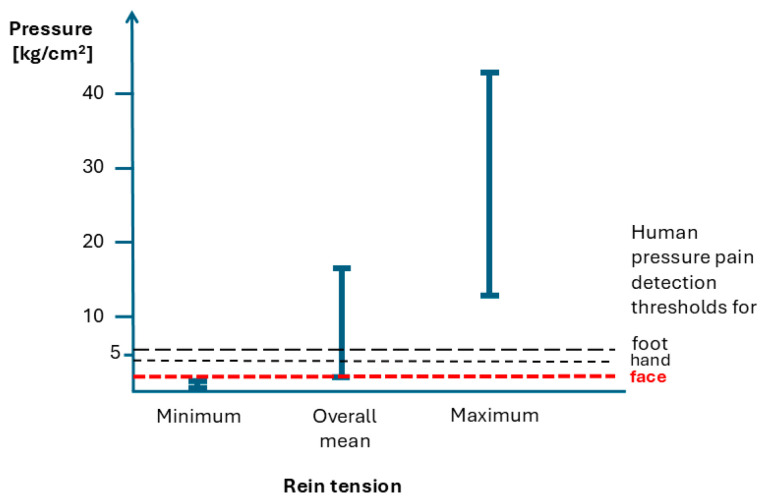
Rein tension in horses and human detection thresholds for pressure pain. Minimal, overall mean, and maximum rein tensions/bit pressures (kg/cm^2^) are shown as vertical lines in accordance with Mellor [10]. The human PPDTs for the face, hand, and foot (kg/cm^2^) are represented as reference lines derived from Magerl et al. [20]. Note that Magerl et al. [20] suggested that pain thresholds for the face are similar for the whole head.

Figure 1 integrates the pressure per unit area associated with rein tension, as reported by Mellor [10], with the human PPDT values for the face, hand, and foot derived from the normative data of Magerl et al. [20]. Rein tensions and bit pressures are presented for the three scenarios: minimum, overall mean, and maximum recorded rein pressure, measured in kilograms per square centimeter (kg/cm^2^) [10]. The human PPDTs for the face, hand, and foot are represented as horizontal lines for comparison.

The figure demonstrates that only the minimum rein tension is below all the human PPDT values presented, indicating that such pressures, if applied to the human face, hand, or foot, would likely not cause pain. However, the majority of these values exceed the human PPDTs and would induce pain at all three locations (face, hand, and foot).

## 3. Stimulus Response Function

An estimate of pain tolerance in humans can be derived from the stimulus response function (SRF). In such experiments, a noxious stimulus is incrementally increased while participants rate their pain using a visual analog scale ranging from “no pain” to “maximum tolerable pain.” This approach establishes the relationship between stimulus intensity and perceived pain. A recent study by Geissler et al. [29] provides these data specifically for pressure-induced pain.

One method to assess pain tolerance in animals is by measuring the withdrawal reflex in response to a noxious stimulus, known as the nociceptive withdrawal reflex (NWR). Mühlemann et al. [30] provided data on horses, where the NWR was elicited through electrical stimulation of the digital nerve, and the withdrawal reflex was recorded via electromyography of the deltoid muscle. The authors further explored the relationship between the intensity of the behavioral response and the level of noxious stimulation, thereby offering (limited) data on the SRF in horses. Although these data pertain to electrical noxious stimulation on the forelimb rather than pressure-induced pain, the SRF derived from these stimuli still offers valuable insights into pain processing in horses. Therefore, we considered the SRF in horses and humans as a translational measure of pain tolerance, trying to assess the intensity of the potentially rein tension and bit pressure associated pain.

The values for human SRF for pressure pain were derived from Geisler et al. [29] (p. 7, Figure 5A). Pressure pain was applied using an algometer, which is also a mandatory tool for QST measurements. The pressure stimuli (probe area: 1 cm^2^) were applied to the tibia bone of the left leg with a steadily increasing intensity of 50 kPa/s.

Study participants reported their pain sensation using a computer-based visual analog scale (VAS), where 0 represented “no pain” and 100 indicated “maximum pain” [29]. Participants were instructed to say “stop” when they reached their maximum pain threshold, at which point the pressure application was immediately halted. To prevent tissue damage, a physical pressure limit of 1500 kPa was enforced.

**Figure 2:**
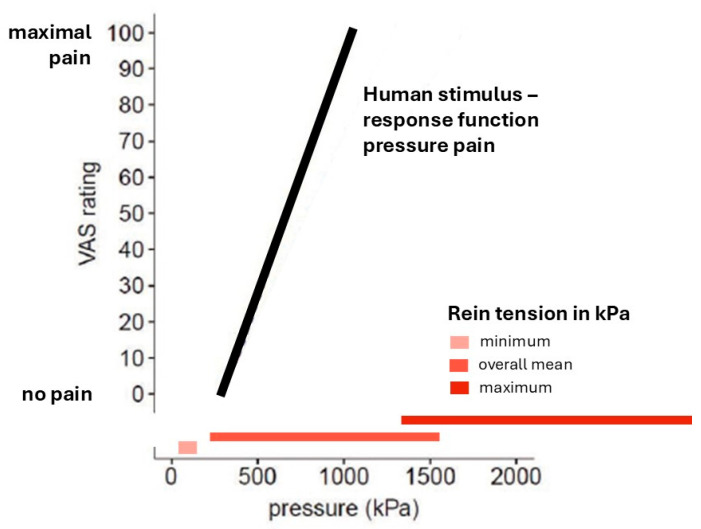
Rein tension in horses and human stimulus response function (SRF) for pressure pain. The human SRF for pressure pain on the tibia bone (adapted from Geisler et al. [29], p. 7, Figure 5A) is shown as a black line in relation to minimal, overall mean, and maximum rein tensions/bit pressures (in kPa), as reported by Mellor [10], represented as red horizontal lines parallel to the *x*-axis.

Figure 2 illustrates how the pressure per unit area values associated with rein tension compared to the human SRF for pressure pain. Data on minimal, overall mean, and maximum rein tensions in relation to the human SRF for pressure pain on the tibia bone are presented. The data indicate that, in the study by Geisler et al. [29], a threefold increase in mechanical pressure shifted pain ratings from the pain detection threshold (VAS 1) to tolerance threshold (usually VAS 100, i.e., the maximum value) on a visual analog scale in humans.

Importantly, the maximum pressure applied in the Geisler et al. [29] study was capped at 1500 kPa for ethical reasons, as higher pressures were expected to cause tissue damage. Notably, the data for the just overall mean rein pressure interval in horses extends up to 1520.03 kPa, while the interval for maximum rein tensions ranges from 1314.09 to 4285.51 kPa. These values exceed the pressure stimuli considered ethically acceptable in human studies by far and reach the threshold for expected tissue damage in humans.

With regard to horses, the data provided by Mühlemann et al. [30] on the NWR and the associated behavioral pain responses suggest that a 1.5-fold increase in pain threshold intensity to electrical stimulation already elicited a maximal behavioral pain response. This is significantly lower than the threefold increase in pain ratings observed in humans in response to pressure pain and may hint that horses reach maximum tolerable pain faster than humans, at least when using electrical painful stimulation.

## 4. Discussion

With the exception of loose reins and the lower range of average rein tension or bit pressure, the levels of pressure commonly applied in equitation, as reported in the scientific literature, are sufficient to cause pain in humans, ranging from mild to severe and even intolerable.

To evaluate the consequences of these findings, it is essential to explore the concepts of pain in greater depth. The term “nociception,” introduced by Charles Scott Sherrington [31], distinguishes the physiological process of nervous activity from the subjective experience of pain [16,32]. As discussed before, nociception is relatively consistent across mammals, but perceived pain and pain thresholds can vary. Moreover, pain detection threshold and pain tolerance are distinct concepts. Pain detection threshold is the minimum stimulus intensity at which pain is first perceived, while pain tolerance is the maximum level of pain an individual can endure before it becomes unbearable. Both concepts are closely linked to how a species’ lifestyle or habitat influences its ability to cope with pain. It has been reasonably suggested that horses, as prey animals, have a high pain tolerance and can endure and manage intense pain. For instance, they are known to suppress signs of discomfort during caretaker visits [11]. Yet, this ability should not be interpreted as horses feeling less pain. As flight animals, they may have simply developed strategies to control pain more efficiently and manage their nociceptive input effectively, particularly to suppress pain in the presence of potential predators.

Since animals cannot verbally communicate pain, it is typically assessed through observed behaviors, such as the nociceptive withdrawal reflex in Mühlemann et al.’s study [30] on horses. Their findings revealed that a 1.5-fold increase in pain threshold intensity from electrical stimulation elicited a maximal behavioral pain response in horses, which is significantly lower than the threefold increase in pain ratings observed in humans under pressure pain [29]. Although differences between electric and pressure-induced pain must be acknowledged, the data suggest that horses may reach their maximum tolerable pain threshold more quickly than humans.

Pressure pain, commonly measured using a device known as an algometer, has been employed in horses and is even recommended as an objective method for pain assessment, because clinical pain evaluations are often subjective, resulting in variability among practitioners in identifying and localizing pain [33,34]. Consequently, using human pressure pain detection thresholds as a foundation for this discussion appears to be well justified. Moreover, using the PPDT of human facial skin as a reference for rein tension-induced bit pressure is most likely a conservative hypothesis and may even underestimate the pain threshold for oral tissues [23].

The question of pressure-induced pain in horses is also directly relevant for bitless bridles, as they typically function by applying pressure to the nasal bone. Studies on noseband tightness reveal that pressure on the nasal bone can be stressful for the horse and even lead to deformations [35,36]. Therefore, it should be noted that bitless bridles, although they may be less likely to cause pain compared to bits, still function by exerting pressure on sensitive facial areas, which could potentially lead to tension-related pain.

The commentary has several limitations: (i) The calculations of rein tension-induced bit pressures are based on the assumption that bits are positioned within a largely toothless region of the gums [10]. While this assumption is well-supported, additional factors, such as the presence of the tongue, may influence these calculations and potentially impact the results. (ii) While we recognize that the complex nature of the phenomenon pain may not allow for a direct comparison between humans and animals—in this case, the horse—we believe that the approach we present here is justifiable given the similarities in physiology and nociception. Pain serves as a protective physiological mechanism, safeguarding the body’s integrity, and is therefore a fundamental function of the nervous system. The ability to detect and respond to aversive environmental stimuli is a core characteristic of animals [13,14]. (iii) While we cannot make definitive statements about how, for example, pain ratings might compare between these two species, it would **not** be in accordance with current physiological knowledge to assume that a stimulus causing considerable to severe pain in one mammal species would not result in pain perception in another. This is particularly true, when tissue damage occurs in response to noxious stimulation, which was expected to occur at about 1500 kPa in the study of Geisler et al. [29] in humans. It seems reasonable to assume that such a stimulus would be perceived as painful across all species. It is important to remember that the widespread use of animals as model species for studying human nociception and pain perception (e.g., Refs. [37,38]) is based on the shared similarities in pain physiology.

While we acknowledge the limitations of our arguments, we believe it is reasonable to consider pain detection thresholds—particularly for pressure—and stimulus response functions as key factors in estimating potential pain in horses. Stimulus response functions have been documented for both species [29,30], making them arguably the most reliable available method for assessing possible pain in horses. However, despite the clear evidence that animals like horses experience pain, they are unable to communicate it directly. Thus, given the complex and multifaceted nature of pain, our calculations should be regarded as estimates rather than definitive measures.

## 5. Conclusions

While the phylogeny of nociception suggests a similar basic physiology for processing of noxious stimuli within the mammal realm, the complex phenomenon of pain perception may vary across species. However, since pain is such a complex phenomenon that includes nociception as well as emotional and behavioral aspects, it cannot be concluded that these three aspects of pain perception are linearly connected for all species. An animal may demonstrate excellent behavioral coping mechanisms in response to pain, but this should not be interpreted as the animal experiencing less pain compared to another that displays signs of pain more openly.

Horses tend to suppress signs of pain and lack vocalizations associated with it. This distinguishes them from other domestic species, such as dogs, which are in this regard more similar to humans. The unique nature of the horses’ pain-related behaviors has been recognized as a significant welfare issue, prompting considerable efforts to improve the diagnosis of pain and associated behaviors in horses (e.g., Refs. [39,40,41,42]).

In conclusion, although direct comparisons between the pain processing of humans and horses may be challenging, we believe that within the framework of the phylogeny of nociception, along with the more complex behavioral and emotional aspects of pain inherent to each species, it is possible to understand the principles of pain processing in horses and relate them to those of the human primate.

Why is this important? As flight animals, horses exhibit fundamental differences in their pain behavior compared to humans. While the challenges to horse welfare in diagnosing chronic pain have been acknowledged over the past decade, the role of acute pain in equitation remains a relatively unexplored area of research. This is further complicated by the practical and ethical challenges associated with investigating the topic. By offering a meta-perspective that integrates aspects of nociception and pain for both species—humans and our companion animal, the horse—we aim to provide a deeper understanding of the role of acute pain in horses.

## Figures and Tables

**Table 1:** Range of bit pressures and rein tensions summarized and reported in Mellor [10], presented in kilopascals (kPa) and kilograms per square centimeter (kg/cm^2^). Note that the values for the category “minimum” intensity are derived from Heleski et al. [28] as cited in Mellor [10].

Rein Tension		
Intensity	kPa	kg/cm^2^
Minimum	91.2–107.87	0.93–1.1
Overall mean	225.55–1520.03	2.3–15.5
Maximum	1314.09–4285.51	13.4–43.7

**Table 2:** Mean human PPDTs for the face, hand, and foot derived from the normative data provided by Magerl et al. [20]. Data are presented in kilopascals (kPa) and kilograms per square centimeter (kg/cm^2^).

Human Pressure Pain Detection Thresholds (PPDTs)		
Location	kPa	kg/cm^2^
Face	232.4	2.37
Hand	445.3	4.54
Foot	535.5	5.47

## Data Availability

No new data were created or analyzed in this study. Data sharing is not applicable.

## Abbreviations

The following abbreviations are used in this manuscript:

PPDT	Pressure pain detection threshold
kPa	Kilopascal
kg/cm^2^	kilograms per square centimeter
N	Newton
QST	Quantitative sensory testing
SRF	Stimulus response function
NWR	Nociceptive withdrawal reflex
